# Validation and comparison of geostatistical and spline models for spatial stream networks

**DOI:** 10.1002/env.2340

**Published:** 2015-04-07

**Authors:** A. M. Rushworth, E. E. Peterson, J. M. Ver Hoef, A. W. Bowman

**Affiliations:** ^1^ School of Mathematics and StatisticsUniversity Gardens, University of GlasgowG12 8QWU.K.; ^2^ Digital Productivity and Services Flagship, Commonwealth Scientific and Industrial Research Organisation (CSIRO)PO Box 2583Brisbane4001QLD; ^3^ NOAA National Marine Mammal Laboratory, Alaska Fisheries Science CenterSeattleWA98115‐6349U.S.A.

**Keywords:** stream network, kriging, P‐splines, semiparametric

## Abstract

Scientists need appropriate spatial‐statistical models to account for the unique features of stream network data. Recent advances provide a growing methodological toolbox for modelling these data, but general‐purpose statistical software has only recently emerged, with little information about when to use different approaches. We implemented a simulation study to evaluate and validate geostatistical models that use continuous distances, and penalised spline models that use a finite discrete approximation for stream networks. Data were simulated from the geostatistical model, with performance measured by empirical prediction and fixed effects estimation. We found that both models were comparable in terms of squared error, with a slight advantage for the geostatistical models. Generally, both methods were unbiased and had valid confidence intervals. The most marked differences were found for confidence intervals on fixed‐effect parameter estimates, where, for small sample sizes, the spline models underestimated variance. However, the penalised spline models were always more computationally efficient, which may be important for real‐time prediction and estimation. Thus, decisions about which method to use must be influenced by the size and format of the data set, in addition to the characteristics of the environmental process and the modelling goals. ©2015 The Authors. *Environmetrics* published by John Wiley & Sons, Ltd.

## 1 Introduction

Large data sets collected on streams and rivers are becoming more common because of broad‐scale environmental‐monitoring programs. These data sets often include measurements such as dissolved pollutant concentrations, stream temperature and measures of biodiversity (i.e. counts of birds and insects) which are collected across the branching stream network. These data are often used to address vital questions pertaining to the effects of climate change on habitat and species distributions, as well as other anthropogenic impacts on instream habitat and aquatic pollution. It is therefore critical that appropriate statistical methods, which adequately account for the different sources of variability, are used to make valid inferences from stream network data.

It is typical to find evidence of residual spatial autocorrelation in modelling settings where the dependent variable of interest is spatially indexed, and this feature is also true of statistical models for stream networks. The residual variation is usually the result of some unobserved confounding variables that are correlated in space and left out of the mean structure which can cause variance parameters to be unreliably estimated. In standard geostatistical approaches, this issue is remedied by including an additional spatial process in the model specification, whose covariance matrix is populated by some appropriate function of the Euclidean separation between pairs of observations.

From a statistical perspective, stream networks have complex spatial characteristics such as the physical structure of the branching network, flow connectivity and flow direction (Peterson *et al*., 2013) that are not easily accounted for with a traditional geostatistical methodology. These characteristics produce discontinuities in physical, chemical and biological properties at stream junctions, where flow mixes and heterogeneous habitat conditions often occur. In addition, stream networks are embedded in the 2‐D terrestrial landscape, which can have a strong influence on instream conditions (e.g. agricultural fields supply excess nutrients to streams via subsurface and overland flow pathways). These unique spatial characteristics produce multiple, multi‐scale patterns of spatial autocorrelation that must be incorporated in the spatial model specification.

A ‘stream distance’ metric, defined as separation measured along the path of the stream, has been used in geostatistics as an alternative to the Euclidean metric (Cressie and Majure, 1997; Gardner *et al*., 2003). However, Ver Hoef *et al*. (2006) show that the use of stream distance within standard geostatistical models does not, in general, yield a valid spatial covariance structure, highlighting the need for more appropriate tools for modelling stream network data. Ver Hoef *et al*. (2006) and Cressie *et al*. (2006) subsequently develop valid covariance models based on moving average constructions integrated over stream distance, which also take into account flow direction between pairs of locations on the stream network and the influence of intervening confluences (i.e. the points at which stream segments converge and flow into a downstream segment). These constructions were further generalised in Ver Hoef and Peterson (2010) to allow models in which spatial dependence propagates upstream as well as downstream, for example, to model the spatial pattern of fish or insects which migrate in either direction. The class of models described in Ver Hoef and Peterson (2010) are supported by the software library SSN (Ver Hoef *et al*., 2014) written in the R language (R Core Team, 2013).

More recently, O'Donnell *et al*. (2014) describe a functional approach to capturing spatial and temporal structure on stream networks, drawing on ideas from the semiparametric modelling literature. Under this framework, the network is partitioned into a set of non‐overlapping stream segments, across each of which the expected response is assumed to be constant. Any fine partition of the stream network may be chosen, but there are conceptual and computational advantages to defining segments that are bounded by confluence points. As a consequence, the expected values at two locations are treated as equivalent unless at least one confluence lies on the path between them. Spatial dependence is then modelled by smoothing across all pairs of segments that are first‐order neighbours of each other. Part of the focus of O'Donnell *et al*. (2014) was in characterising non‐linear temporal change and non‐separable space‐time structure, and doing so in a manner that avoids the computational difficulty that can hamper a geostatistical approach. The methods described in O'Donnell *et al*. (2014) are available in the R package smnet (Rushworth,).

The geostatistical methods first proposed by Ver Hoef *et al*. (2006) and later generalised by Ver Hoef and Peterson (2010) and the penalised spline models developed by O'Donnell *et al*. (2014) are relatively recent methodological developments (hereafter referred to as Ver Hoef and Peterson (VHPT) and O'Donnell (OD), respectively). In addition, both methods are fairly accessible to the scientific community via open source software. The contribution that this study makes is to provide a validation of these two inferential frameworks, and to highlight similarities and differences in the two approaches. For example, the model of OD is inherently discrete in its representation of a stream network which can result in smaller computational overheads for large data. In contrast, the model of VHPT assumes an underlying network representation that is continuous, which enables an understanding of fine scale behaviour of the underlying spatial process. This difference in network representation means that the discrete approach of OD can be treated as approximating the more detailed approach of VHPT, and so this study also aims to provide an understanding of the extent to which the spatial attributes of the data may impede or enhance this approximating behaviour. To satisfy these aims, we undertake a comparison in which both models are fitted to simulated data under the VHPT models with a broad range of spatial structures. We also provide practical guidance about the relative costs and benefits of the two approaches in terms of model fit and computational overhead.

The paper is structured as follows: Section 2 provides a summary of the theoretical aspects of the two approaches being compared, while Section 3 describes the design and implementation of a comprehensive simulation study, which provides insight into the variability in model performance across a range of realistic, simulated stream network data. In Section 4, the resulting performances of each model are evaluated and the main features and differences are summarised. Some discussion follows in Section 5 about the implications of the main findings for practitioners, with suggestions concerning the future development of both methodologies.

## 2 Geostatistical Models

In this section, the stream network modelling approaches of Ver Hoef *et al*. (2006) and O'Donnell *et al*. (2014) are described in greater detail before the comparison study of Section 3 is discussed.

### 2.1 Geostatistical models

#### 2.1.1 Moving average construction

As discussed in Section 1, Ver Hoef *et al*. (2006) show that the use of a standard geostatistical model, using stream distance as a separation metric, does not generally result in valid covariance structures except in the case where the exponential covariance function is used. As a result, Ver Hoef *et al*. (2006) and Cressie *et al*. (2006) seek to build appropriate covariance structures via the use of moving average constructions. This construction states that a random variable *Z* can be defined as the convolution of a moving average function *g* and a white‐noise process *W* so that

(1) $$Z(s|\theta )=\overset{\infty }{\underset{-\infty }{\int }}g(x-s|\theta )\mathrm{dW}(x)$$

where *x* and *s* are locations on the real line. *Z* then has covariance defined in terms of the choice of moving average function *g*:

(2) $$\text{Cov}(Z(s),Z(s+h))=C(h|\theta )=\overset{\infty }{\underset{-\infty }{\int }}g(x|\theta )g(x-h|\theta )\mathrm{dx}$$

where *h* is a distance metric such as Euclidean distance. Because the the argument *x* is defined on the real line in Equations (1) and (2), some additional work is required to adapt the process *Z* and *g* to the context of a stream network, which is a set of connected line segments embedded in $R^{2}$. This domain is more complex than Euclidean space, and it is necessary to establish a terminology for stream network topology, point locations and separation metrics; for consistency with previous authors, we adopt a similar nomenclature to that used in Ver Hoef *et al*. (2006).

#### 2.1.2 Terminology

**Figure 1:**
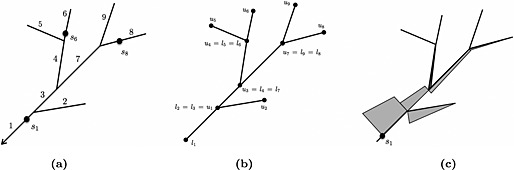
(a) is an example of an enumeration of the stream segments in a stream network, with locations shown on stream segments using the notation *s*_∙_, direction of flow is indicated by the arrowhead; (b) is an illustration of the naming conventions for the beginning and end points for each stream segment; and (c) provides a visual representation of a moving average function where the height of each rhombus corresponds to the relative size of the function and decreases with distance from the example location *s*_1_

First, an enumeration of each of the segments of a single stream network *i*∈*I*={1,2,…,*n*_seg_} is defined, where a segment is defined as the stretch of stream between branching or confluence points on the network (Figure 1(a)). Point locations on a stream network cannot be uniquely identified using upstream distance alone, and requires additional information about the branch of the stream network on which the point lies. The notation *x*_*i*_ can therefore be used to uniquely identify points, where *x* is the stream distance separating the most downstream location on the stream network (i.e. outlet) and the point, while *i* identifies the stream segment on which the point lies. The most downstream location on the *i* ^th^ stream segment is called *l*_*i*_, while the most upstream location is called *u*_*i*_, shown in Figure 1(b). It is then useful to define the set of stream segments upstream of, and including a location *i*, which we call *U*_*i*_⊆*I*, while the set that excludes *i* is called $U_{i}^{*}\subseteq I$. In a similar fashion, the set of stream segments downstream of, and including *i* is called *D*_*i*_⊆*I*, and $D_{i}^{*}\subseteq I$ if the set excludes *i*. This notation is necessary to formally define the concept of ‘flow‐connectivity’, where water flows from an upstream location to a downstream location. Specifically, *U*_*i*_∩*U*_*j*_≠*∅* implies that the stream segments *i* and *j* are *flow‐connected*, and conversely if *U*_*i*_∩*U*_*j*_=*∅* then *i* and *j* are *flow‐unconnected*. Note that if two segments do not reside on the same stream network, then they are neither flow‐connected nor flow‐unconnected. These definitions are also required to specify the valid covariance models over the spatial stream network that are described next in Section 2.1.3.

#### 2.1.3 Tail‐up model

A stream network can be represented as a collection of connected line segments, and adapting the moving average construction to this setting requires the integral in Equation (2) to be performed in a piece‐wise manner over these. An important feature of a stream network is the influence of flow direction on spatial dependence; as such, Ver Hoef *et al*. (2006) chose moving average functions *g*(*x*|***θ***) that are defined only upstream of a given location *x*. Furthermore, Ver Hoef *et al*. (2006) recognise that spatial dependence on a stream network may be influenced by the relative flow volumes that converge at confluence points, which can be incorporated into the definition of the moving average function by dividing *g* at confluences relative to the proportions associated with the contributing stream segments. This results in a scaling of the new segment‐wise integral by a weight *ω*_*k*_

(3) $$Z(s_{i}|\theta )=\overset{u_{i}}{\underset{s_{i}}{\int }}g(x_{i}-s_{i}|\theta )\mathrm{dW}(x_{i})+\underset{j\in U_{i}^{*}}{\sum }\left(\underset{k\in B_{i,j}}{\prod }\sqrt{\omega_{k}}\right)\overset{u_{j}}{\underset{l_{j}}{\int }}g(x_{j}-s_{i}|\theta )\mathrm{dW}(x_{j})$$

where *B*_*i*,*j*_=*D*_*j*_∩*D*_*i*_. If a confluence upstream of segment *i* has upstream segments *j* and *k*, then 0≤*ω*_*j*_,*ω*_*k*_≤1 and *ω*_*j*_+*ω*_*k*_=1. A visual representation of a moving average function is provided in Figure 1(c), which shows how the moving average function ‘splits’ at confluences, and how locations farther upstream have little influence on *s*_1_. To avoid truncating the moving average function, terminal stream segments (those furthest upstream) are treated as having infinite length. The construction in Equation (3) implies non‐zero covariance between *Z*(*s*) and *Z*(*s* + *h*) if they are flow‐connected, which is particularly desirable when the observed data are strongly dependent on flow, as may be the case with the concentrations of dissolved pollutants. Ver Hoef *et al*. 2006) show that Equation ((3) implies a covariance between a pair of locations (*r*_*i*_,*s*_*j*_) that is defined as

(4) $$C_{u}(r_{i},s_{j}|\theta )=\left\{\begin{array}{cc} \pi_{i,j}C_{t}(h|\theta ) & \;\text{if}r_{i}<s_{j}\;\text{are flow‐connected}\\ 0 & \;\text{if}r_{i}\text{and}s_{j}\;\text{are flow‐unconnected} \end{array}\right.$$

where *h* is the stream distance separating *r*_*i*_ and *s*_*j*_, and $\pi_{i,j}=\underset{k\in B_{i,j}}{\prod }\sqrt{\omega_{k}}\in [0,1]$ represents the influence of the spatial weights on the covariance between flow‐connected *r*_*i*_ and *s*_*j*_. Equations (3) and (4) define a model that is referred to as a ‘Tail‐up’ model by Ver Hoef and Peterson (2010), in order to distinguish it from moving average constructions that permit non‐zero covariance between flow unconnected locations, known as ‘Tail‐down models’. Tail‐down models are not considered in this manuscript, but more can be found in Ver Hoef and Peterson (2010). Different choices are available for the moving average function *g*, resulting in a process, *Z*, with different covariance properties. For example, the exponential moving average function is defined as

(5) $$g(x|\theta )=\theta_{1}\exp(-x/r_{\Phi })I(0\leq x)$$

where *r*_Φ_ is a range parameter. The moving average function in Equation (5) yields the unweighted covariance function

$$C_{t}(h|\theta )=\theta_{v}\exp(-h/r_{\Phi })$$

where *θ*_*v*_ is the ‘partial sill’ parameter and is a function of *θ*_1_ and *r*_Φ_. Other choices for the moving average function and their associated covariance functions are described in Ver Hoef and Peterson (2010).

When covariate information relevant to the spatial response is available at each observation location, these can be modelled as linear fixed effects within the framework proposed by Ver Hoef and Peterson (2010), alongside the spatial processes described previously. Estimation of the linear terms and covariance function parameters is achieved using restricted maximum likelihood (REML).

### 2.2 Penalised model

A recent development by O'Donnell *et al*. (2014) describes a model for stream network data using a flexible regression framework, which draws on ideas from the semiparametric modelling literature. By first defining a non‐overlapping spatial partition of the spatial stream network, O'Donnell *et al*. (2014) treat a network as a set of connected line segments. Although many such partitions are possible, the simplest and most convenient consists of the set of stream segments that are bounded only by confluence points and the most upstream and downstream network locations. This definition coincides with that used by VHPT in Section 2.1.2 for stream segments, and the same segment enumeration, {1,2,…,*n*_seg_}, can be used for simplicity.

**Figure 2:**
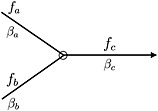
Schematic representation of a confluence, with model parameters (*β*_*a*_, *β*_*b*_), flow volumes (*f*_*a*_, *f*_*b*_) and the corresponding outgoing versions (*β*_*c*_, *f*_*c*_). The black circle represents the point of confluence and the arrow represents the direction of flow

Having selected a partition, a parameter representing the expected value of the response variable is associated with each stream segment, denoted by *β*_*i*_ where *i*∈{1,2,…,*n*_seg_}. This enables a regression model for data **y** = (*y*_1_,…,*y*_*n*_)^⊤^ collected on the stream network to be constructed:

(6) y=Bβ+ϵ

where ***ϵ*** ∼ *N*(**0**,*σ* ^2^ ***I***), $\beta ={\left(\beta_{1},\ldots ,\beta_{n_{\text{seg}}}\right)}^{⊤}$ and **B** is an *n* × *n*_seg_ indicator matrix whose *i*th row takes the value 1 in the column corresponding to the stream segment upon which *y*_*i*_ was observed and 0 elsewhere. Since there are typically fewer data points than parameters ***β***, and spatial autocorrelation is likely to be present in the response variable, ***β*** is penalised so that the parameters in Equation (6) can be estimated. This is achieved by penalising ‘roughness’ over first order neighbourhoods of stream segments, where the roughness measure takes account of network attributes such as relative flow volumes and network topology. To illustrate, an idealised set of stream segments that are joined at a confluence point are shown in Figure 2.

Using the example in Figure 2, the mean value of a response variable at the downstream segment *c* can be thought of as being determined by the mean value at the upstream locations *a* and *b*, whose respective contributions depend on the relative volumes of flow each contributes to *c*. Denoting these flow volumes by *f*_*a*_, *f*_*b*_, *f*_*c*_, and assuming that *f*_*c*_=*f*_*a*_+*f*_*b*_, the concept of mass balance implies that

(7) $$\begin{array}{cc} \frac{f_{a}}{f_{c}}\beta_{a}+\frac{f_{b}}{f_{c}}\beta_{b} & =\beta_{c}\\ \frac{f_{a}}{f_{c}}(\beta_{a}-\beta_{c})+\frac{f_{b}}{f_{c}}(\beta_{b}-\beta_{c}) & =0 \end{array}$$

Equation (7) gives rise to a natural roughness measure for *β*_1_,…,*β*_*p*_, given by

(8) $$\frac{f_{a}^{2}}{f_{c}^{2}}{\left(\beta_{a}-\beta_{c}\right)}^{2}+\frac{f_{b}^{2}}{f_{c}^{2}}{\left(\beta_{b}-\beta_{c}\right)}^{2}$$

which describes a measure of deviation from Equation (7). Estimating the simple regression model in Equation (6) involves minimising a sum of squared deviations. In a similar manner, a model that penalises ***β*** according to Equation (8) can be estimated by minimising the appended objective function

(9) $${\left(y-B\beta \right)}^{⊤}(y-B\beta )+\lambda \underset{a,b∼c}{\sum }\left(\frac{f_{a}^{2}}{f_{c}^{2}}{\left(\beta_{a}-\beta_{c}\right)}^{2}+\frac{f_{b}^{2}}{f_{c}^{2}}{\left(\beta_{b}-\beta_{c}\right)}^{2}\right)$$

(10) $$={\left(y-B\beta \right)}^{⊤}(y-B\beta )+\lambda \beta^{⊤}D^{⊤}D\beta$$

Equation (9) uses *a*,*b* ∼ *c* to signify that stream segments *a* and *b* are connected to *c* through a mutual confluence point, and the associated sum is over all such stream segment triplets. Equation (10) presents the same expression in matrix notation where the matrix **D** represents a difference matrix in which each row has two non‐zero elements in column locations corresponding to two adjacent stream segments, for example, $\frac{f_{a}}{f_{c}}$ and $-\frac{f_{b}}{f_{c}}$. It is not clear how much influence the roughness penalty should have over the estimation of ***β***, and so a smoothness control parameter *λ* is included in Equation (9) to modulate the strength of this influence. For fixed *λ* and normal errors, ***β*** can then be estimated by $\hat{\beta }={\left(B^{⊤}B+\lambda D^{⊤}D\right)}^{-1}B^{⊤}y$. Since different values of *λ* can imply different levels of goodness of fit, a value must be found that strikes a balance between extremes of model complexity. This is achieved in O'Donnell *et al*. (2014) by minimising a model performance criterion such as the corrected Akaike Information Criterion (AICC of Hurvich *et al*. (1998) which is defined as $\log(\hat{\sigma })+(2\mathrm{df}+2)/(n-\mathrm{df}-2)$, where *df* represents the degrees of freedom of the model.

The models of VHPT and OD take different approaches to characterising spatial structure; VHPT populates a covariance matrix using functions of flow, catchment information and stream distance, whereas OD construct sparse penalty matrix using the same characteristics. The penalty matrix of OD is analogous to the sparse precision matrices used in Gaussian Markov random field (GMRF) prior distributions for areal data in which pairs of non‐adjacent spatial units are assumed to be conditionally independent. There is therefore a connection to the literature in ordinary geostatistics that compares the use of GMRF as an approximation to a Gaussian random field (see for example Song *et al*. (2008)).

In addition to providing a novel way of capturing spatial structure on a stream network, O'Donnell *et al*. (2014) describe extensions that embed such a stream network model within a spatio‐temporal regression framework that allows a flexible functional representation of the potentially non‐linear effects of covariates. These extensions permit the fitting of a powerful class of models, but for reasons of brevity are not considered within the current study. Instead, the focus is placed on the properties and performance of the stream network model only.

## 3 Simulation study design

Our goal was to validate and compare the performance and reliability of the models of OD and VHPT over a realistic range of simulated data. Although there are many aspects of stream network data that may impact model performance, we focused primarily on the density of spatial observations, number of stream segments, different types of spatial covariance structures under which data are generated and the effects of different strengths of linear covariates. To be as relevant as possible to the typical goals of a spatial analysis, we emphasised spatial predictive accuracy and coverage, and also the estimation of linear terms.

### 3.1 Simulating stream network branching structure

**Figure 3:**
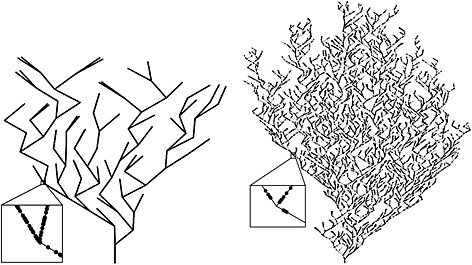
Plots depicting the two particular network structures on which all of the simulated data were generated: on the left is the smaller network with *n*_seg_=100 stream segments and on the right is the large network with *n*_seg_=2000 stream segments. The prediction locations are shown by black points on each network, magnified examples of which are shown in the insets

In order to compare both of the approaches described in Section 2, it was important to simulate data with structures similar to those encountered in typical stream networks such as large numbers of segments that are very sparsely sampled. Both of these attributes are likely to have a substantial impact on model performance and were therefore a key component of the study design. The SSN software package can be used to construct dendritic stream network structures with a pre‐specified number of stream segments, nseg, which enabled the investigation of the impact of small (nseg = 100) and large (nseg = 2000) networks. Note that the simulated networks do not contain bisecting or diverging segments. Constructing valid dendritic stream networks with several hundred or more branches was computationally intensive, and so this operation was performed only once at the outset, and these two network structures were fixed for the duration of the study (Figure 3).

We investigated several factors that impacted model performance. We used sample sizes of *n* = 100 and *n* = 500 spatial design points whose locations were generated randomly and independently across each of the two networks, and these were also held fixed for the study duration. Our overall goal was to validate two stream network models by investigating their performance of estimation and prediction, and so an additional fixed set of 1000 prediction locations were generated in the same fashion for both large and small networks.

### 3.2 Spatial correlation structure of simulated data

In order to build appropriate spatial covariance into data that was simulated at the locations shown in Figure 3, the spatial process defined by the exponential Tail‐up model (TU) described in 2.1 was used. Other covariance functions could have been used within the class of TU models, but to maintain a feasible number of different factors in the simulation experiment, the effect of generating data from different covariance functions was not investigated. Since this choice of spatial structure comes from the class of TU models, absolute model fit performance would favour the closely related VHPT model under study. However, we were primarily concerned with validation and in understanding the approximating ability of the OD approach to VHPT, rather than direct and objective comparison of the two modelling approaches. Therefore, the use of a TU process to generate data should not erode the generality of any findings. We also investigated the impact of response variables that exhibited Euclidean spatial dependence in addition to TU‐type dependence across the stream network. This was important because spatial structure in stream network data can arise as the result of a mixture of processes, some of which occur on the network and others in the terrestrial landscape within which the network is embedded. For a fixed set of network locations ***s*** = (*s*_1_,…,*s*_*n*_)^⊤^ with corresponding Cartesian coordinates ***C*** = [***x*** ^⊤^,***y*** ^⊤^], the process *Y* was simulated as Gaussian with mean depending on covariates {*X*_1_,*X*_2_,*X*_3_}, and a set of spatial processes {*Z*_1_,*Z*_2_}, representing TU and Euclidean structures, respectively. *Y* can be expressed as

(11) $$\begin{array}{ccc} Y(s)|Z_{1},Z_{2}∼ & \;N\left(\beta_{0}1^{⊤}+k_{1}\overset{3}{\underset{i=1}{\sum }}\beta_{i}X_{i}(s)+Z_{1}(s)+k_{2}Z_{2}(s),\sigma^{2}I\right)\\ Z_{1}(s)∼ & \;N\left(0,\Phi \right)Z_{2}(s)∼ & \;N\left(0,\Psi \right) \end{array}$$

(12) $$\begin{array}{cc} \Phi_{\mathrm{ij}}= & \;\left\{\begin{array}{cc} \pi_{\mathrm{ij}}\exp\left(-\frac{\left|s_{i}-s_{j}\right|}{r_{\Phi }}\right) & \;\text{if}s_{i}\text{and}s_{j}\text{are flow‐connected,}\\ 0 & \;\text{otherwise} \end{array}\right.\\ \Psi_{\mathrm{ij}} & =\exp\left(-\frac{\left||c_{i}-c_{j}|\right|}{r_{\Psi }}\right) \end{array}$$

where |*s*_*i*_−*s*_*j*_| was the stream distance between locations *s*_*i*_ and *s*_*j*_, *r*_Φ_ and *r*_Ψ_ were range parameters for each spatial process and *π*_*ij*_ was a set of weights determined by the number and influence of branches between locations *s*_*i*_ and *s*_*j*_. ***c***_*i*_ represents the *i*th row of ***C***. The processes {*X*_1_}, {*X*_2_} and {*X*_3_}, involved in the linear component $\beta_{0}1^{⊤}+\overset{3}{\underset{i=1}{\sum }}\beta_{i}X_{i}(s)$, were each based on TU spatial structures, to simulate spatially patterned covariate effects; these were simulated with a spatial range of *v*/2 where *v* was the maximum separation between points on the network, a nugget effect of 0.1 and a partial sill of 1. Different realisations of {*X*_1_}, {*X*_2_} and {*X*_3_} were used for each scenario of the simulation study. To simulate the effect of observing a variable that was unrelated to the response variable, we set *β*_3_=0. The remaining variables {*X*_1_} and {*X*_2_} were set to have a significant association with *Y* with coefficients *β*_0_=*β*_1_=*β*_2_=1. In order to simulate unobserved confounding, *X*_2_(***s***) was assumed unobserved and was not included in model fitting.

We varied the spatial components (TU and Euclidean), fixed effects and covariance parameters, as well as the number of network segments and observations (Table 1) to generate a total of 32 different simulation scenarios. The Euclidean component, *Z*_2_, was specified by a fixed range of *r*_Ψ_=0.3*v* and a partial sill of 1. The binary control parameter *k*_2_ denoted the presence (*k*_2_=1) or absence (*k*_2_=0) of Euclidean spatial structure in the data‐generating process for a given simulation scenario. For the TU component, *Z*_1_, the range parameter *r*_Φ_ was set to take two possible values, {0.3*v*,*v*}, in order to simulate spatial network structures with both long and short range dependence. The role of parameter *k*_1_ was to scale the strength of the spatial component relative to the linear component and was given the values {0.1,1}. All TU and Euclidean components require nugget and partial sill parameters that we fixed at 0.1 and 1, respectively. The variance parameter *σ* ^2^ was also fixed at a value of 0.1.

**Table 1:** Parameter levels used in the simulation study

Parameter	Levels	Interpretation
*k*_1_	{0.1,1}	Strong/weak linear effect
*k*_2_	{0,1}	Presence/absence of Euclidean structure
*r*_Φ_	{0.3*v*,*v*}	Long/short range tail‐up structure
*n*	{100,500}	Small/large number of observations
*n*_seg_	{100,2000}	Small/large network

### 3.3 Model fitting and measuring performance

In addition to fitting the models of VHPT and OD, it was also desirable to compare each model's relative performance to some baseline model. This model could have taken a number of forms, but for simplicity, a standard, non‐spatial, linear regression model was used. The three models that were fitted to the vector of observations *Y*(***s***), when only the TU spatial structure was simulated were

(13) $$\text{VHPT}\;\;\beta_{0}1^{⊤}+\beta_{1}X_{1}(s)+\beta_{3}X_{3}(s)+Z_{1}(s)+\epsilon$$

(14) $$\text{OD}\;\;\beta_{0}1^{⊤}+\beta_{1}X_{1}(s)+\beta_{3}X_{3}(s)+B\beta_{s}+\epsilon$$

(15) $$\text{Linear}\;\;\beta_{0}1^{⊤}+\beta_{1}X_{1}(s)+\beta_{3}X_{3}(s)+\epsilon$$

where ***ϵ*** is independent *N*(**0**,*σ* ^2^ ***I***) and *Z*_1_ was a TU spatial process with unknown sill (*θ*) and range parameters (*r*) as described in Section 2.1. The spatial component ***B*** *β*_*s*_ in Equation (14) was constructed from an *n* × *n*_seg_ binary stream segment membership matrix and vector of *n*_seg_ spatial parameters, as outlined in Section 2.2. When Euclidean spatial dependence was present in addition to TU, the following appended models were fitted:

(16) $$\text{VHPT}\;\;\beta_{0}1^{⊤}+\beta_{1}X_{1}(s)+\beta_{3}X_{3}(s)+Z_{1}(s)+Z_{2}(s)+\epsilon$$

(17) $$\text{OD}\;\;\beta_{0}1^{⊤}+\beta_{1}X_{1}(s)+\beta_{3}X_{3}(s)+B\beta_{s}+m(x,y)+\epsilon$$

(18) $$\text{Linear}\;\;\beta_{0}1^{⊤}+\beta_{1}X_{1}(s)+\beta_{3}X_{3}(s)+\epsilon$$

where Equation (16) includes an additional Euclidean spatial *Z*_2_ process with exponential covariance function and unknown range and sill parameters. Similarly, the OD model in Equation includes a bivariate smooth term $m(x,y)=B(x,y)\gamma^{\prime }=\left(B(x)⊗1_{n_{\text{seg}}}\right)⊙\left(1_{n_{\text{seg}}}⊗B(y)\right)$ where **B**(**y**) and **B**(**x**) were B‐spline basis matrices each with *n*_seg_ knots; $\left[x^{⊤},y^{⊤}\right]$ correspond to the vector of network locations ***s*** transformed back to Cartesian coordinates, and ***γ*** was a vector of basis coefficients also estimated by penalised least squares, where smoothness was controlled by a single control parameter.

To obtain a broad understanding of model performance, we measured the predictive performance and the ability to estimate the fixed effects parameters. The former was estimated using root‐mean‐squared prediction error (RMSPE), estimation bias and 90% prediction interval coverage, while for the latter, root‐mean‐squared error (RMSE), estimation bias and 90% confidence interval coverages were used. To make accurate comparisons, the observed data were generated 500 times from each simulation scenario. Then, summary statistics for interval coverage, bias and RMSE were obtained from all 500 model fits. All of the simulation described was performed in under 48h using a 2 × Quad Core Intel Xeon CPU X5570 clocked at 3.0GHz with 8MB Cache and 32Gb RAM. Furthermore, the models of VHPT were fitted using the R package SSN which benefited from the Intel^®;^ Math Kernel Library (Intel, 2012), which provides advanced linear algebra routines for parallelising intensive operations such as matrix inversion.

We also wanted to learn about the computational efficiency of each model. Therefore, we measured the time it took to fit the VHPT (Equation (13)) and OD (Equation (14)) models for multiple network sizes (*n*={50,100,200,500,1500}) and numbers of data points (*n*_seg_={50,100,200,500,1500}). Since network size and number of data points are likely to be the main factors that drive computational complexity, simulated data were generated from the model described in Section 3.2 with parameters *k*_1_=0.1, *k*_2_=0 and *r*_Φ_=*v* held fixed across all network and data size scenarios. For each of the network sizes and number of data points, 500 data sets were simulated, and the time taken to fit each of the models was recorded.

## 4 Results

We now present the results of the simulation study by evaluating the predictive performance in Section 4.1 and the results of fixed effects estimation in Section 4.2. For each of prediction and fixed effects performance, and for data simulated with tail‐up only and tail‐up with Euclidean structure, plots of bias, squared error and interval coverage were generated for the three models.

### 4.1 Predictive performance

**Figure 4:**
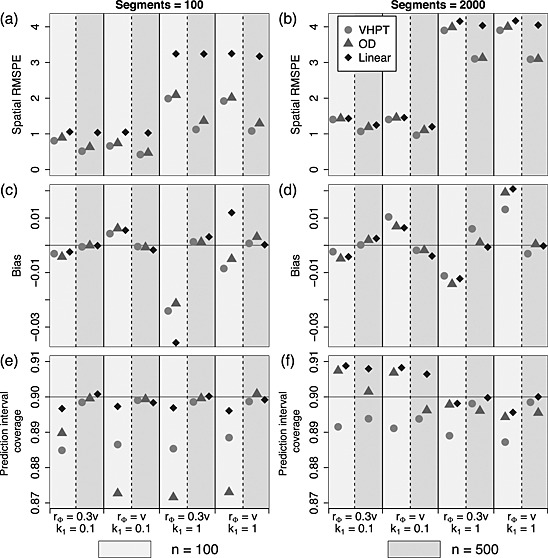
Predictive performance summaries for Ver Hoef and Peterson, O'Donnell and linear models fitted to data with tail‐up spatial structure, where the models correspond to those shown in Equations (13), (14) and (15). Values for *n* = 100 are shown to the left (light shaded rectangles) and *n* = 500 to the right (dark shaded rectangles) above each parameter combination. (a) and (b) show the relative root‐mean‐squared prediction error for each technique, (c) and (d) show the bias and (e) and (f) show the prediction interval coverage. The four *x*‐axes index the different choices made in fixing the spatial structure of the simulated data

**Figure 5:**
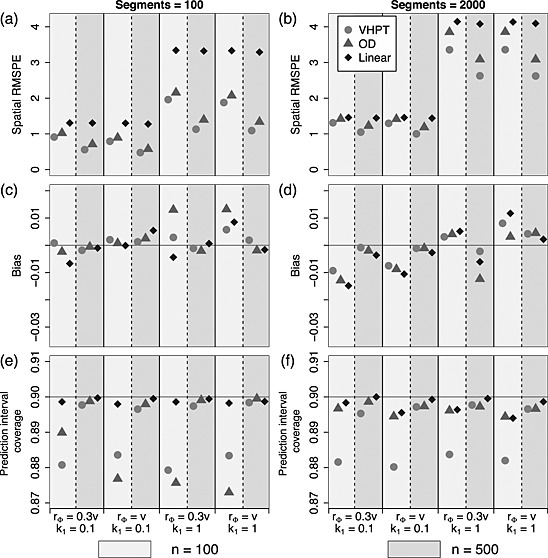
Predictive performance summaries for Ver Hoef and Peterson, O'Donnell and linear models fitted to data with tail‐up and Euclidean mixture spatial structure, where the models correspond to those shown in Equations (16), (17) and (18). Values for *n* = 100 are shown to the left (light shaded rectangles) and *n* = 500 to the right (dark shaded rectangles) above each parameter combination. (a) and (b) show the relative root‐mean‐squared prediction error for each technique, (c) and (d) show the bias and (e) and (f) show the prediction interval coverage. The four *x*‐axes index the different choices made in fixing the spatial structure of the simulated data

Broadly similar patterns were visible across the different simulated spatial structures; the lowest RMSPE was associated with data exhibiting a long spatial range (*r*_Φ_=0.30) and a weak linear component (*k*_1_=0.1), while the highest RMSPE was associated with short spatial range and a dominant linear component. In terms of RMSPE, shown in Figures 4(a) and (b) and 5(a) and (b), the spatial models outperformed the linear model in all scenarios, and of these, VHPT performed slightly better than OD. Under the most sparsely sampled scenario (*n*_seg_=2000, *n* = 100), the prediction error was almost equivalent for each of the models (Figure 4(a) and (b)).

In general, prediction bias was highest overall under the smallest sample size (*n* = 100; Figure 4(c) and (d)). Similar patterns were visible for all three models in the TU only scenario where the linear model performed only slightly more poorly than the others. When the Euclidean component was present, the relative performances (Figure 5(c) and (d))were less easily interpreted; although VHPT clearly achieved a lower level of bias than OD and the linear model. However, the bias was always relatively small compared with RMSPE, and so all estimates could be considered unbiased.

When the number of observations was highest (*n* = 500) the empirical interval coverages were generally close to the nominal level of 90% (Figures 4(e) and (f) and 5(e) and (f)). However, under sparse sampling of the small network (*n* = 100, *n*_seg_=100), slightly lower coverages between 0.87 and 0.89 were found for VHPT and OD. It was notable that for the model of VHPT, this feature was only visible when *n*_seg_=2000.

### 4.2 Fixed effects estimation

**Figure 6:**
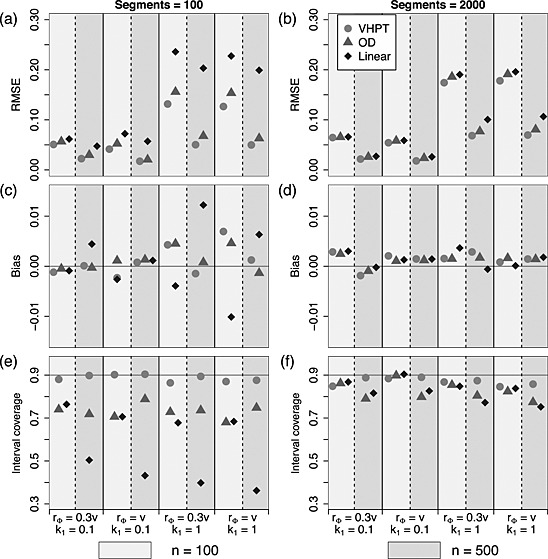
Summaries of estimation performance for Ver Hoef and Peterson, O'Donnell and linear models fitted to data with tail‐up spatial structure, where the models correspond to those shown in Equations (13), (14) and (15). Values for *n* = 100 are shown to the left (light shaded rectangles) and *n* = 500 to the right (dark shaded rectangles) above each parameter combination. (a) and (b) show the relative root‐mean‐squared error for each technique in estimating the linear parameters, (c) and (d) show the bias and (e) and (f) show the confidence interval coverage. The four *x*‐axes index the different choices made in fixing the spatial structure of the simulated data

**Figure 7:**
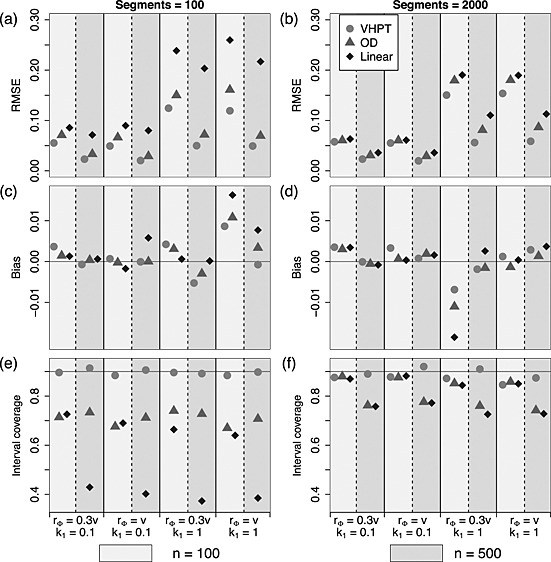
Summaries of estimation performance for Ver Hoef and Peterson, O'Donnell and linear models fitted to data with tail‐up and Euclidean mixture spatial structure, where the models correspond to those shown in Equations (16), (17) and (18). Values for *n* = 100 are shown to the left (light shaded rectangles) and *n* = 500 to the right (dark shaded rectangles) above each parameter combination. (a) and (b) show the relative root‐mean‐squared error for each technique in estimating the linear parameters, (c) and (d) show the bias and (e) and (f) show the confidence interval coverage. The four *x*‐axes index the different choices made in fixing the spatial structure of the simulated data

In order to summarise the ability of each model to estimate the fixed effect parameters, and for reasons of brevity, the results shown in Figures 6 and 7 are average across the parameters *β*_1_ and *β*_3_. The intercept term *β*_0_ is omitted here because, in most spatial analyses, it is not usually of direct scientific interest. Similar patterns in RMSE to those in RMSPE were found across each simulated spatial scenario (Figures 6(a) and (b) and 7(a) and (b)), where VHPT performs the best, closely followed by OD. In contrast, the linear model performs relatively poorly. An exception occurs under the sparsest sampling scenario (*n* = 100, *n*_seg_=2000), where the error rates were essentially equivalent. Figures 6(c) and (d) and 7(c) and (d) show that bias was low overall, and was roughly similar for each of the three models. As with prediction bias, the estimation bias was low relative to RMSE and indicates that these were unbiased estimates.

Figures 6e and 7e show that the empirical coverages for the models of VHPT and OD differed markedly for the smallest network (*n*_seg_=100); regardless of spatial structure and *n*, VHPT achieved the nominal 90%, while OD coverages were between 70% and 80%. For larger networks, the coverages occupied a narrower range of values around the target, and VHPT were closest to the 90%. Interestingly, OD achieved better coverage when fewer data points were present (*n* = 100).

### 4.3 Computation

**Figure 8:**
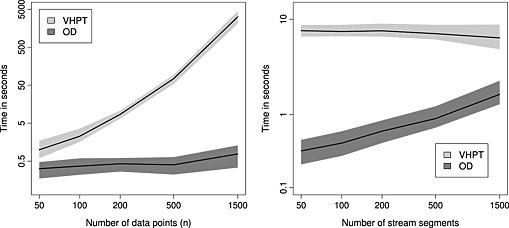
Plots showing how computation time scales with number of data points *n* (left panel) and with number of stream segments *n*_seg_ (right panel). The shaded regions contain the computation times between the upper 95% percentile and the lower 5% percentile, where the dark grey regions correspond to the models of O'Donnell and the light grey regions to Ver Hoef and Peterson

In addition to the empirical properties of estimation and prediction, we compared the relative computation times for each of the spatial models. The time taken for the VHPT model increases rapidly with *n*, whereas larger models can be fit with OD relatively quickly (left panel of Figure 8). The time taken to fit the model of VHPT remained roughly constant across *n*_seg_, while for the model of OD, the time to fit increases with network size; although the time taken was still less than under VHPT (right panel of Figure 8).

## 5 Discussion

In this study, two different spatial statistical approaches used to model stream network data were compared across a wide variety of simulated data. We acknowledge that our results may be somewhat limited because the fitted TU model of VHPT was the true model for the simulated data, and that other spatial structures may be encountered in practice. However, the decision to simulate from the TU covariance model reflects the type of spatial structures that have been found in practice (Peterson *et al*., 2006; Isaak *et al*., 2010; Ruesch *et al*., 2012; McGuire *et al*., 2014), and because the SSN software was a convenient way to simulate these otherwise highly complex structures. Consequently, our aim was to investigate the performance of each model and the extent to which the discrete approach of OD could approximate that of VHPT. In addition, we wanted to provide potential users with recommendations about which model is appropriate for their data and goals.

Our results showed that the size of the network and sampling density affected VHPT and OD model estimation, predictive ability and computational efficiently differently. The moving average approach of VHPT generally outperformed OD in terms of prediction and estimation in densely sampled stream networks because it offers a more flexible and realistic description of spatial dependence within a segment when data contain information about fine scale dependence. Thus, when accurate estimation of a fixed effect is required, or when predictions are required when the spatial coverage of the stream network is more complete, the models of VHPT would likely be a more appropriate choice. However, OD achieved almost equivalent performance to VHPT when the ratio of stream segments to data points was large and the data were consequently less informative about fine scale dependence. In these cases, OD also required much lower computational effort. These features give the OD model an advantage in many real‐world situations such as high‐frequency temporal sampling with relatively poor spatial coverage, or where increased computational efficiency is needed for real‐time prediction and estimation. Not surprisingly, both spatial models outperformed a linear model that assumed independent errors, in terms of prediction and estimation, except when there were a small number of observations. Thus, when data exhibit spatial dependence, either spatial model would be preferable to the linear model that assumes independent errors.

Decisions about which method to use should also be influenced by the type of observations, the characteristics of the environmental process being modelled and the functionality of the supporting software. For example, the SSN package can fit *generalised* linear spatial models with a variety of commonly used link functions, and permits the fitting of mixtures of spatial covariance structures including the Euclidean, TU and recently developed ‘tail‐down’ construction (Ver Hoef and Peterson, 2010). On the other hand, the methodology described by O'Donnell *et al*. (2014) can be used to capture non‐linear functional relationships between covariates. As such, smnet allows the user to fit spatial additive models based on P‐splines, which may be particularly useful for capturing non‐stationary and non‐separable spatio‐temporal effects; characteristics that we expect to be common in streams data (Peterson *et al*., 2013). Until recently, fitting these types of stream network models would have required a great deal of effort and technical expertise, but the SSN and smnet packages make these methods accessible to modellers from a wide variety of disciplines. The two packages also use the same data structure for storing the essential attributes of a stream network, which enables modellers to easily explore their data using a variety of methods that more adequately represent fundamental stream processes. The results of this study provide guidance to statisticians, stream ecologists and natural resource managers about which of these relatively new methods are most suitable for their data sets and goals.

## References

[env2340-bib-0001] Cressie N , Frey J , Harch B , Smith M . 2006 Spatial prediction on a river network. Journal of Agricultural, Biological, and Environmental Statistics 11(2):127–150.

[env2340-bib-0002] Cressie N , Majure JJ . 1997 Spatio‐temporal statistical modeling of livestock waste in streams. Journal of Agricultural, Biological, and Environmental Statistics 2:24–47.

[env2340-bib-0003] Gardner B , Sullivan PJ , Lembo AJ, Jr. . 2003 Predicting stream temperatures: geostatistical model comparison using alternative distance metrics. Canadian Journal of Fisheries and Aquatic Sciences 60(3):344–351.

[env2340-bib-0004] Hurvich CM , Simonoff JS , Tsai CL . 1998 Smoothing parameter selection in nonparametric regression using an improved Akaike information criterion. Journal of the Royal Statistical Society: Series B (Statistical Methodology) 60:271–293.

[env2340-bib-0005] Intel . 2012 Intel math kernel library 11.0.

[env2340-bib-0006] Isaak DJ , Luce CH , Rieman BE , Nagel DE , Peterson EE , Horan DL , Parkes S , Chandler GL . 2010 Effects of climate change and wildfire on stream temperatures and salmonid thermal habitat in a mountain river network. Ecological Applications 20(5):1350–1371. 20666254 10.1890/09-0822.1

[env2340-bib-0007] McGuire KJ , Torgersen CE , Likens GE , Buso DC , Lowe WH , Bailey SW . 2014 Network analysis reveals multiscale controls on streamwater chemistry. Proceedings of the National Academy of Sciences 111(19):7030–7035. 10.1073/pnas.1404820111 PMC4024884 24753575

[env2340-bib-0008] O'Donnell D , Rushworth A , Bowman AW , Marian Scott E , Hallard M . 2014 Flexible regression models over river networks. Journal of the Royal Statistical Society: Series C (Applied Statistics) 63(1):47–63. 10.1111/rssc.12024 PMC4303988 25653460

[env2340-bib-0009] Peterson EE , Merton AA , Theobald DM , Urquhart NS . 2006 Patterns of spatial autocorrelation in stream water chemistry. Environmental Monitoring and Assessment 121(1):569–594. 10.1007/s10661-005-9156-7 16897525

[env2340-bib-0010] Peterson EE , Ver Hoef JM , Isaak DJ , Falke JA , Fortin MJ , Jordan CE , McNyset K , Monestiez P , Ruesch AS , Sengupta A , Som N , Steel EA , Theobald DM , Torgersen CE , Wenger SJ . 2013 Modelling dendritic ecological networks in space: an integrated network perspective. Ecology Letters 16(5):707–719. 23458322 10.1111/ele.12084

[env2340-bib-0011] R Core Team . 2013 R: A language and environment for statistical computing. R Foundation for Statistical Computing, Vienna, Austria.

[env2340-bib-0012] Ruesch AS , Torgersen CE , Lawler JJ , Olden JD , Peterson EE , Volk CJ , Lawrence DJ . 2012 Projected climate‐induced habitat loss for salmonids in the John Day river network, Oregon, USA. Conservation Biology 26(5):873–882. 22827880 10.1111/j.1523-1739.2012.01897.x

[env2340-bib-0013] Rushworth A . 2014 2014. smnet: Smoothing for stream network data. R package version 1.0.

[env2340-bib-0014] Song H‐R , Fuentes M , Ghosh S . 2008 A comparative study of Gaussian geostatistical models and Gaussian Markov random field models. Journal of Multivariate Analysis 99(8):1681–1697. 19337581 10.1016/j.jmva.2008.01.012 PMC2662683

[env2340-bib-0015] Ver Hoef JM , Peterson EE . 2010 A moving average approach for spatial statistical models of stream networks. Journal of the American Statistical Association 105(489):6–18.

[env2340-bib-0016] Ver Hoef JM , Peterson EE , Clifford D , Shah R . 2014 SSN: An R package for spatial statistical modeling on stream networks. Journal of Statistical Software 56(3.

[env2340-bib-0017] Ver Hoef JM , Peterson EE , Theobald D . 2006 Spatial statistical models that use flow and stream distance. Environmental and Ecological Statistics 13(4):449–464 (English).

